# Supplementary material for: Modifications of Chest CT Body Composition Parameters at Three and Six Months after Severe COVID-19 Pneumonia: A Retrospective Cohort Study
Source: Nutrients. 2022 Sep 13;14(18):3764. doi: 10.3390/nu14183764 (PMC9501258; doi:10.3390/nu14183764)
Supplement: Supplementary file 1 [file nutrients-14-03764-s001.zip › nutrients-1840809-supplementary.pdf]

Supplementary materials

Supplementary Table S1

	Mean (SD)		
	**T0**	**T1**	**T2**
**SAT – observed only (n=138)**	159.8 (81.3)	156.3 (89.8)	161.1 (101.6)
**SAT – estimated only (n=65)**	289.6 (130.7)	297.3 (143.4)	291.9 (140.9)

Supplementary Table S1: Observed and visually estimated TAT and SAT at each time point.

Supplementary Table S2

	**Baseline**		**T1**	
	coefficient	p-value	coefficient	p-value
**TAT – BMI (n=132)**	0.70	<0.0001	0.73	<0.0001
**SAT – BMI (n=132)**	0.64	<0.0001	0.68	<0.0001
**TAT – SAT (n=202)**	0.97	<0.0001	0.98	<0.0001

Supplementary Table S2: Linear association coefficients at baseline and T1 (2-3 months after diagnosis) among BMI, body mass index, TAT, total adipose tissue area, and SAT, subcutaneous adipose tissue area.

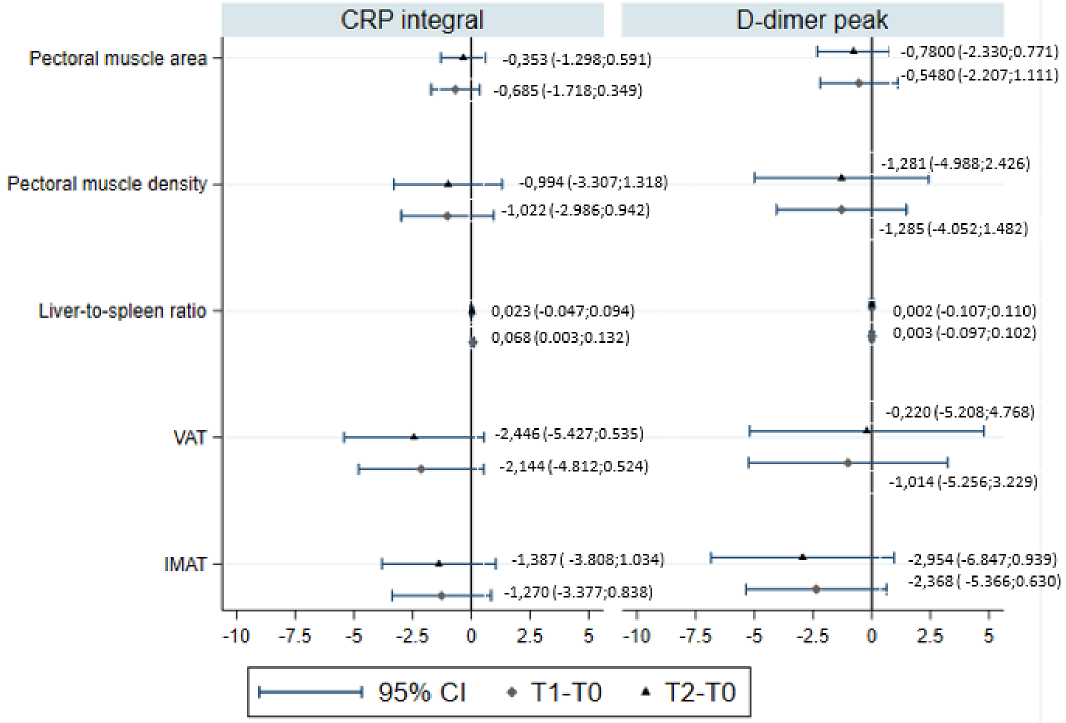
CRP integral
D-dimer peak
Pectoral muscle area
Pectoral muscle density
Liver-to-spleen ratio
VAT
IMAT
-0,353 (-1.298;0.591)
-0,685 (-1.718;0.349)
-0,994 (-3.307;1.318)
-1,022 (-2.986;0.942)
0,023 (-0.047;0.094)
0,068 (0.003;0.132)
-2,446 (-5.427;0.535)
-2,144 (-4.812;0.524)
-1,387 (-3.808;1.034)
-1,270 (-3.377;0.838)
-0,7800 (-2.330;0.771)
-0,5480 (-2.207;1.111)
-1,281 (-4.988;2.426)
-1,285 (-4.052;1.482)
0,002 (-0.107;0.110)
0,003 (-0.097;0.102)
-0,220 (-5.208;4.768)
-1,014 (-5.256;3.229)
-2,954 (-6.847;0.939)
-2,368 (-5.366;0.630)
-10 -7.5 -5 -2.5 0 2.5 5
-10 -7.5 -5 -2.5 0 2.5 5
95% CI
T1-T0
T2-T0

A

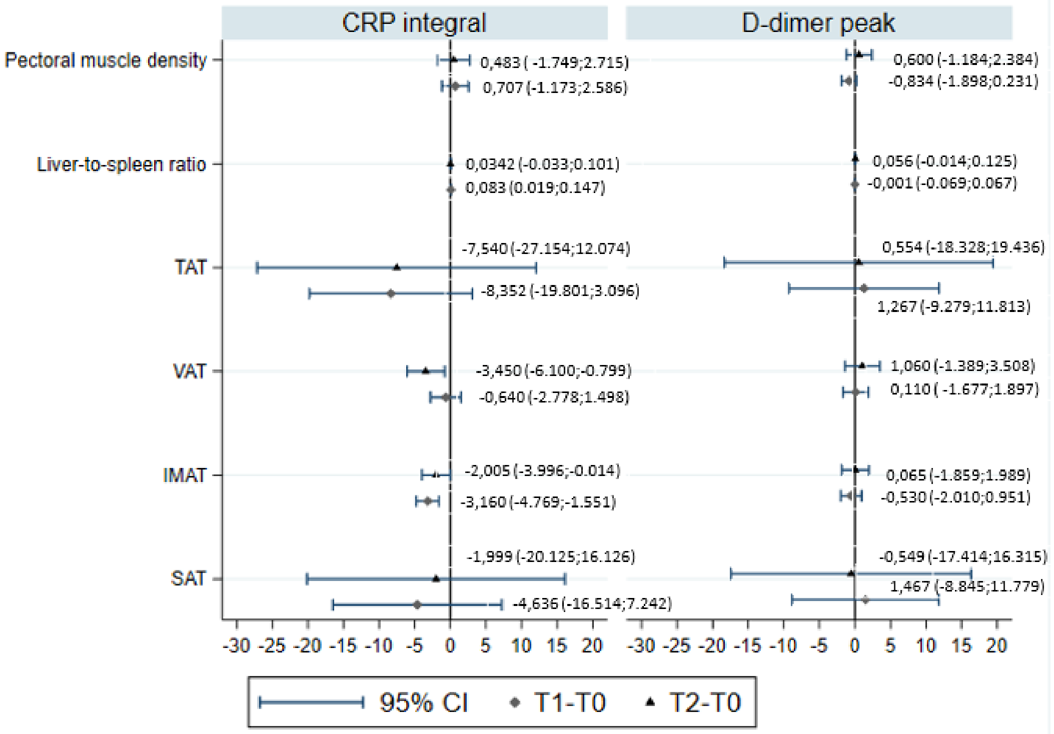
CRP integral
D-dimer peak
Pectoral muscle density
Liver-to-spleen ratio
TAT
VAT
IMAT
SAT
0,483 (-1.749;2.715)
0,707 (-1.173;2.586)
0,0342 (-0.033;0.101)
0,083 (0.019;0.147)
-7,540 (-27.154;12.074)
-8,352 (-19.801;3.096)
-3,450 (-6.100;-0.799)
-0,640 (-2.778;1.498)
-2,005 (-3.996;-0.014)
-3,160 (-4.769;-1.551)
-1,999 (-20.125;16.126)
-4,636 (-16.514;7.242)
0,600 (-1.184;2.384)
-0,834 (-1.898;0.231)
0,056 (-0.014;0.125)
-0,001 (-0.069;0.067)
0,554 (-18.328;19.436)
1,267 (-9.279;11.813)
1,060 (-1.389;3.508)
0,110 (-1.677;1.897)
0,065 (-1.859;1.989)
-0,530 (-2.010;0.951)
-0,549 (-17.414;16.315)
1,467 (-8.845;11.779)
-30 -25 -20 -15 -10 -5 0 5 10 15 20
-30 -25 -20 -15 -10 -5 0 5 10 15 20
95% CI
T1-T0
T2-T0

B

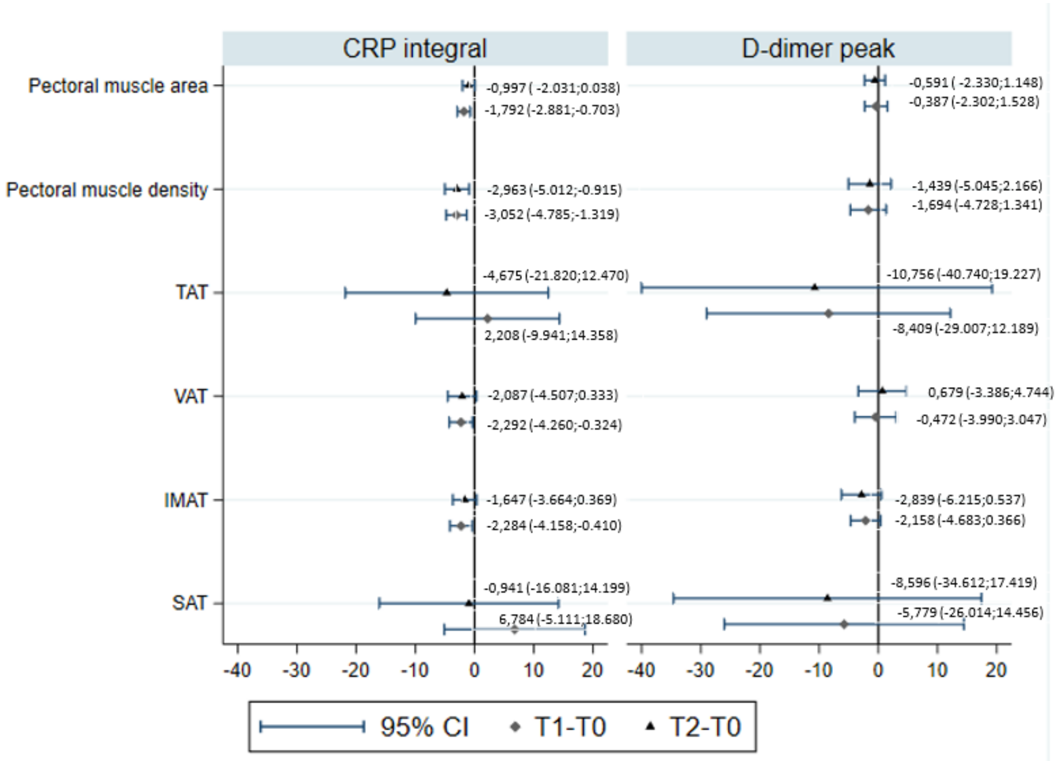

C

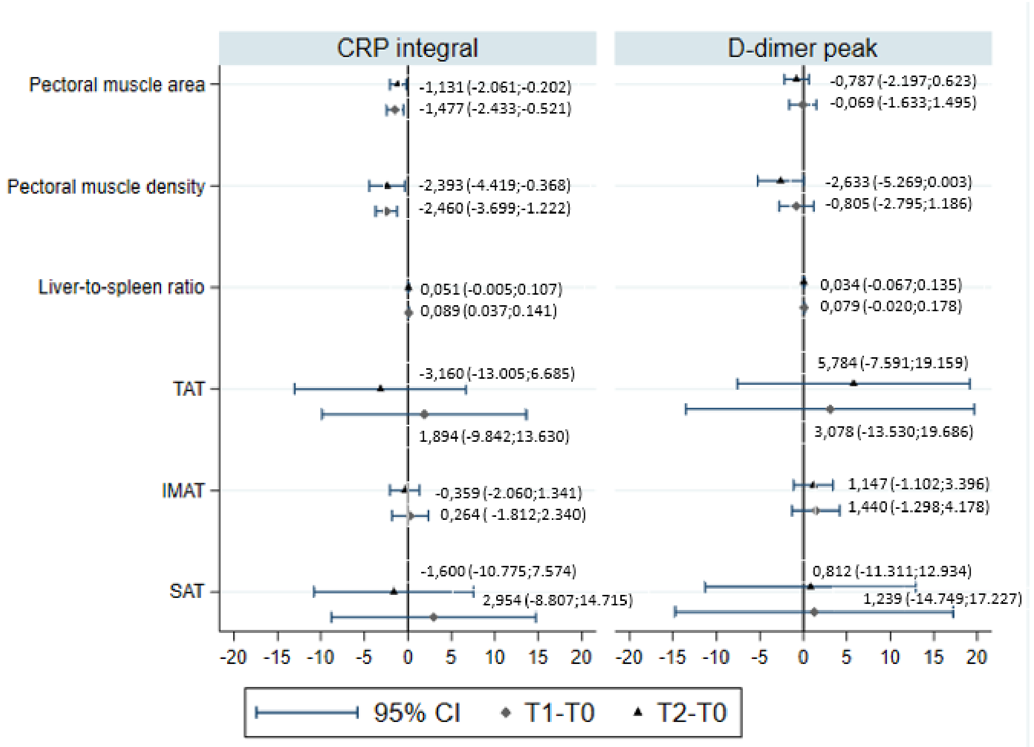

D

Supplementary Figure S1: Associations of CRP integral and D-dimer peak with CT body composition changes in models adjusted for age, sex, and baseline TAT, in different subgroups of patients: highest TAT tertile (A), lowest pectoral muscle area tertile (B), lowest liver-to-spleen ratio tertile (C), and highest VAT tertile (D).
